# Heatwave-associated *Vibrio* infections in Germany, 2018 and 2019

**DOI:** 10.2807/1560-7917.ES.2021.26.41.2002041

**Published:** 2021-10-14

**Authors:** Thomas Theo Brehm, Laura Berneking, Meike Sena Martins, Susann Dupke, Daniela Jacob, Oliver Drechsel, Jürgen Bohnert, Karsten Becker, Axel Kramer, Martin Christner, Martin Aepfelbacher, Stefan Schmiedel, Holger Rohde, the German Vibrio Study Group, German Vibrio Study Group, German Vibrio Study Group, Veronika Balau, Elsa Baufeld, Simone Brechmann, Lutz Briedigkeit, Stephan Diedrich, Ulrike Ebert, Helmut Fickenscher, Bojana Grgic, Claus-Dieter Heidecke, Peter Hinz, Ada Hoffmann, Meike Holbe, Ralf Ignatius, Olaf Kaup, Martin Kern, Martina Kerwat, Ingo Klempien, Georg Lamprecht, Astrid Meerbach, Alexander Mischnik, Andreas Podbielski, Stephan Schaefer, Roman Schwarz, Eckhard Strauch, Philipp Warnke, Simone Weikert-Asbeck, Maria Witte, Waleed Zaki

**Affiliations:** 1 Division of Infectious Diseases, I. Department of Internal Medicine, University Medical Center Hamburg-Eppendorf, Hamburg, Germany; 2 German Center for Infection Research (DZIF), Partner Site Hamburg-Lübeck-Borstel-Riems, Hamburg, Germany; 3 These authors contributed equally to this article and share first authorship; 4 Institute of Medical Microbiology, Virology and Hygiene, University Medical Center Hamburg-Eppendorf, Hamburg, Germany; 5 Institut für Meereskunde, Centrum für Erdsystemwissenschaften und Nachhaltigkeit, University Hamburg, Hamburg, Germany; 6 Robert Koch Institute, ZBS 2: Centre for Biological Threats and Special Pathogens, Highly Pathogenic Microorganisms, Berlin, Germany; 7 Robert Koch Institute, MF1: Bioinformatics, Berlin, Germany; 8 Friedrich Loeffler-Institute of Medical Microbiology, University Medicine Greifswald, Greifswald, Germany; 9 Institute of Hygiene and Environmental Medicine, University Medicine Greifswald, Greifswald, Germany; 10 The members of the Study Group are listed at the end of the article

**Keywords:** Vibriosis, *Vibrio vulnificus*, *Vibrio cholerae*, *Vibrio parahaemolyticus*, Baltic Sea, climate change

## Abstract

**Background:**

*Vibrio* spp. are aquatic bacteria that prefer warm seawater with moderate salinity. In humans, they can cause gastroenteritis, wound infections, and ear infections. During the summers of 2018 and 2019, unprecedented high sea surface temperatures were recorded in the German Baltic Sea.

**Aim:**

We aimed to describe the clinical course and microbiological characteristics of *Vibrio* infections in Germany in 2018 and 2019.

**Methods:**

We performed an observational retrospective multi-centre cohort study of patients diagnosed with domestically-acquired *Vibrio* infections in Germany in 2018 and 2019. Demographic, clinical, and microbiological data were assessed, and isolates were subjected to whole genome sequencing and antimicrobial susceptibility testing.

**Results:**

Of the 63 patients with *Vibrio* infections, most contracted the virus between June and September, primarily in the Baltic Sea: 44 (70%) were male and the median age was 65 years (range: 2–93 years). Thirty-eight patients presented with wound infections, 16 with ear infections, six with gastroenteritis, two with pneumonia (after seawater aspiration) and one with primary septicaemia. The majority of infections were attributed to *V. cholerae* (non–O1/non-O139) (n = 30; 48%) or *V. vulnificus* (n = 22; 38%). Phylogenetic analyses of 12 available isolates showed clusters of three identical strains of *V. vulnificus*, which caused wound infections, suggesting that some clonal lines can spread across the Baltic Sea.

**Conclusions:**

During the summers of 2018 and 2019, severe heatwaves facilitated increased numbers of *Vibrio* infections in Germany. Since climate change is likely to favour the proliferation of these bacteria, a further increase in *Vibrio*-associated diseases is expected.

## Introduction

The genus *Vibrio*, which belongs to the family *Vibrionaceae* and the class *Gammaproteobacteria*, includes many species that are potential human pathogens [1]. *V. cholerae* is a highly diverse species that consists of more than 200 serogroups [2]. Strains within the serogroups O1 and O139 produce cholera toxin and are the causative agents of endemic and epidemic cholera, which represent an important cause of morbidity and mortality in countries with inadequate access to clean water and sanitation facilities [3]. *V. cholerae* strains not included in these serogroups as well as other *Vibrio* spp. are referred to as non-cholera *Vibrio* spp. and are ubiquitous aquatic bacteria with a worldwide distribution, especially in warm estuarine and marine ecosystems [2]. These halophilic bacteria prefer low to moderate salinity (less than 25 parts per thousand (ppt) NaCl) [4]. The abundance of *Vibrio* spp. in marine and estuarine waters closely corresponds with the sea surface temperatures (SSTs) since they proliferate in warm water [5]. Thus, regional variations in environmental conditions are paramount importance in understanding the ecology of *Vibrio* spp.

Human infections with non-cholera *Vibrio* spp. can manifest as wound infections, ear infections, gastroenteritis, and primary septicaemia and have been predominantly reported in tropical and subtropical regions [6]. In Europe, cases are rare, and infections associated with the Mediterranean Sea [7-9], the Atlantic Ocean [10-12], or the Baltic Sea [13,14] have only been sporadically reported. However, a rapidly warming marine environment accompanied by an increase in extreme weather events such as heatwaves has resulted in unprecedented peak SSTs favouring the spread of *Vibrio* spp. worldwide. Recently, larger *Vibrio* spp. outbreaks have been reported in temperate regions such as Spain [15], Sweden, and Finland [16]. In Germany, *Vibrio* spp. other than toxigenic *V*. *cholerae* strains were not classified as reportable pathogenic agents before March 2020; to date, only a few cases have been reported and few case series of autochthonous infections have been published [17-20]. Over the last decades, resistance to various antibiotics, including to ampicillins, tetracyclines, and carbapenems, has emerged among *Vibrio* spp [21,22]. As a result of resistant isolates, the monitoring of antibiotic profiles of clinical and environmental *Vibrio* spp. has become of increasing importance. However, information on the number of human cases and the abundance of *Vibrio* spp. in coastal waters is scarce in most other European countries.

When we became aware of a high number of human infections with virulent *Vibrio* strains contracted in the Baltic Sea during the summer months of 2018 and 2019 (data not shown), we decided to conduct an observational retrospective multi-centre cohort study of domestically-acquired *Vibrio* infections. This study describes the epidemiology and the clinical impact of those infections and assesses antibiotic resistance patterns and phylogenetic relationships among clinical isolates. To determine the association between *Vibrio* infections and SST anomalies, we analysed the degree of warming in the south-western Baltic Sea, where the majority of the infections occurred.

## Methods

### Study population

Although non-cholera *Vibrio* spp. were not notifiable pathogenic agents in Germany before March 2020, clinical isolates of *V. cholerae* could be submitted to the Unit of Highly Pathogenic Microorganism (ZBS 2) at the Center for Biological Threats and Special Pathogens at the Robert Koch Institute (RKI), Germany’s national public health institute, for confirmation and serotyping. In addition, clinical isolates of other virulent *Vibrio* spp. could be sent for further analysis to the consultant laboratory for *Vibrio* spp. at the Federal Institute for Risk Assessment (BfR).

To identify as many patients with *Vibrio*-associated diseases as possible, the first author contacted the RKI, the BfR, and hospitals and microbiological laboratories within 20 km of the German North Sea or Baltic Sea coastline. Patients with *Vibrio* infections and a recent history of foreign travel who presumably did not acquire those infections in Germany were not included in the analysis. We (TTB, LB, HR) developed a standardised case report form, which was used to obtain demographic and clinical characteristics of patients from the treating hospitals and physicians.

### Laboratory investigations

Antibiotic susceptibility testing and whole genome sequencing (WGS) of *V. cholerae* isolates initially submitted to the Unit of Highly Pathogenic Microorganisms (ZBS 2) at the Centre for Biological Threats and Special Pathogens at the RKI by microbiological laboratories or hospitals were performed directly at the RKI. All other isolates were analysed at the Institute of Medical Microbiology, Virology and Hygiene at the University Medical Center Hamburg-Eppendorf.

Species isolates were confirmed by matrix-assisted laser desorption ionization/time-of-flight mass spectrometry (MALDI-TOF) fingerprinting using a biotyper instrument (Bruker Daltonics, Bremen, Germany). To verify a *V. cholerae* species-specific sequence of *sodB* gene (superoxide dismutase), multiplex real-time PCR (5´ nuclease assay) was performed on all suspected isolates. The same assay confirmed the lack of *ctxA*, the encoding cholera toxin produced by toxigenic *V. cholerae*. In addition, conventional PCRs for the determination of serotype serogroup-specific gene *rfb* were performed to confirm the identification of non-O:1/non-O:139 *V. cholerae* strains. Primers and probes were used as previously described [23].

### Antibiotic susceptibility testing

Available *Vibrio* isolates were subjected to antimicrobial susceptibility testing (AST) using disk diffusion and classified according to Clinical and Laboratory Standards Institute (CLSI) breakpoints [24]. Briefly, *Vibrio* isolates were grown on Colombia sheep blood agar at 37 °C for 18 hours. A colony suspension equivalent to 0.5 McFarland was prepared in 0.85% NaCl, streaked on Müller Hinton agar (MHA) plates, and incubated with respective antimicrobial agent disks at 35 ± 2 °C for 18 hours. Sixteen types of antibiotics disks (Oxoid by Thermo Fischer Scientific, Waltham, USA) were used: ampicillin (10 μg), amoxicillin-clavulanic acid (20/10 μg), piperacillin-tazobactam (100/10 μg), amikacin (30 μg), cefepime (30 μg), cefuroxime (30 μg), cefotaxime (30 μg), ceftazidime (30 μg), gentamicin (10 μg), imipenem (10 μg), meropenem (10 μg), ciprofloxacin (5 μg), levofloxacin (5 μg), chloramphenicol (30 μg), sulfamethoxazole/trimethoprim (1.25/23.75 μg), and tetracycline (30 μg). *Escherichia coli* ATCC 25922 was included as a positive control in each test. After incubation, the inhibition zone was measured and interpreted based on guidelines of CLSI M45-A2 [24]. Results for disk diffusion testing are shown in Supplementary Table S1.

### Whole genome sequencing

WGS was performed retrospectively with an Illumina NextSeq instrument (Illumina, San Diego, USA) using 2 x 150 bp paired-end chemistry. Sequencing reads have been deposited in NCBI’s small reads archive (BioProject: PRJNA723758). Processing of WGS data for further phylogenetic analysis was performed at the Institute of Medical Microbiology, Virology and Hygiene at the University Medical Center Hamburg-Eppendorf. Reads were assembled with shovill 1.0.4 (https://github.com/tseemann/shovill) and spades 3.13.1 [25] and annotated with prokka 1.13.3 [26]. An average nt identity over 80% with genomes of the respective reference genome strains (Vibrio fluvialis_ASM155841v2; Vibrio parahaemolyticus_ASM19609v1; Vibrio vulnificus_ASM221513v1; Vibrio cholera ASM674v1; and Vibrio alginolyticus ASM35417v2) confirmed the identified species [27]. Sequencing reads were initially mapped to the respective reference genome and variants were called using snippy (https://github.com/tseemann/snippy). An alignment of core genome single nucleotide polymorphisms (SNPs) was produced in snippy to infer a phylogeny. The phylogenetic tree was constructed with Molecular Evolutionary Genetic Analysis (MEGA) X version 10.2.0 [28] using a neighbour-joining algorithm [29]. Possible relationships between clusters, potential genotypic, and phenotypic traits associated with pathogenicity and source of isolation were investigated. The virulence gene profile was determined with ABRicate (https://github.com/tseemann/abricate) and an additional blast-search against the virulence factor database [30].

### Climatological analyses

SST data were originally derived using the near-infrared channel of the space-borne Advanced Very High Resolution Radiometer (AVHRR) satellite. We used the Reynolds Optimum Interpolation Sea Surface Temperature (OISST) data set with a spatial resolution of ca 25 km. The daily data were obtained from National Oceanic and Atmospheric Administration’s (NOAA) National Center for Environmental Information (NCEI) [31]. The annual climatological cycle from 1982 to 2001 was calculated using the OISST as a reference. SST anomalies were calculated from the higher resolving Moderate Resolution Imaging Spectroradiometer (MODIS) SST data, which have been available since 2002 from the MODIS sensors onboard the EOS-AQUA and Terra satellites. The MODIS Aqua SST data (version 2019 Reprocessing, NASA OB.DAAC, Greenbelt, USA) were provided with a time variable by the Integrated Climate Data Center (ICDC) at the University of Hamburg, Germany. The MODIS sensors offer a spatial resolution of 4.6 km; however, because the sensors cannot penetrate clouds, the data coverage is reduced. The annual climatology from the OISST data was interpolated to the higher resolution of the MODIS data and subtracted from the MODIS SST time series. In addition, a daily MODIS SST spatial average was calculated for the German coastlines where the infections in our study population occurred. The trend in SSTs was calculated from the low-passed SSTs (19 months) by a simple linear regression using standard statistics. Only values with a significance of p < 0.05 were considered.

### Ethical statement

The study was reviewed and approved by the Ethics Committee of the Medical Council of Hamburg (PV7066) and written informed consent was waived because of the retrospective design.

## Results

### Patients and course of disease

Of the 63 patients with domestically-acquired *Vibrio* infections, 44 (70%) were male and the median age for all patients was 65 years (range: 2–93 years). Reliable anamnestic information about the precise place of infection was available for 36 patients, all of whom contracted *Vibrio* infections after recreational exposure to the open Baltic Sea or its estuaries (n = 34) or after consumption of shrimp caught in the North Sea or the Baltic Sea (n = 2). Nine patients were diagnosed while hospitalised near the Baltic Sea, so it is highly likely that their infections occurred during exposure to the Baltic Sea. For the remaining 18 patients, the place of infection is not known. A total of 38 patients (60%) presented with wound infections, 16 (25%) with ear infections, and six (10%) with gastroenteritis (Table 1).

**Table 1:** Characteristics of patients with domestically-acquired *Vibrio* spp. infections by age group, Germany, 2018 and 2019 (n = 63)

Characteristic	Total (n = 63)	Wound infections (n = 38)	Ear infections (n = 16)	Gastroenteritis (n = 6)	Pneumonia (n = 2)	Primary septicaemia (n = 1)
Age in years, Median (range)	65 (2–93)	69 (7–93)	13 (3–78)	60 (2–88)	73 (68–78)	88
**Sex**						
Male	44	26	10	5	2	1
Female	19	12	6	1	0	0
***Vibrio* spp.**						
*V. cholerae* *(non-O1/non-O139)*	30	9	15	5	0	1
*V. vulnificus* ^a^	24	22	0	0	2	0
*V. parahaemolyticus* ^a^	5	4	1	0	0	0
*V. fluvialis*	3	2	0	1	0	0
*V. alginolyticus*	2	2	0	0	0	0
**Comorbidities**						
Information available	41	33	5	1	2	0
Chronic disease	25	22	0	1	2	0
Immunosuppression^b^	8	6	0	1	1	0
**Course of disease**						
Information available (n)	41	33	5	1	2	0
Antibiotic treatment	37	32	2	1	2	0
Hospital admission	35	31	1	1	2	0
Intensive care unit admission	21	18	0	1	2	0
Death	8	6	0	1	1	0

^a^ One patient was co-infected with *V. vulnificus* and *V. parahaemolyticus*, which were detected in wounds as well as in blood cultures.

^b^ Immunosuppression was the result of immunosuppressive therapy, underlying haematological malignancies, or splenectomy.

**Figure 1:**
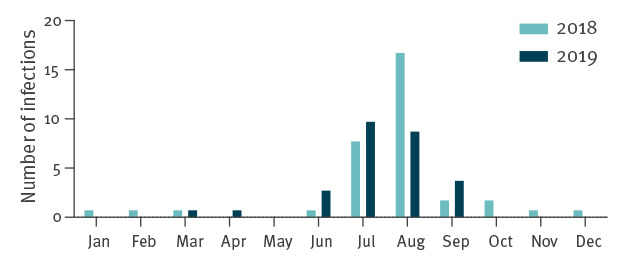
Monthly distribution of patients with domestically-acquired *Vibrio* spp. infections, Germany 2018 and 2019

The vast majority of infections occurred in the summer months between June and September (n = 54; 86%) (Figure 1). In addition, nine infections with *V. cholerae* (non-O1/non-O139) occurred between October and April. Among these cases, detailed information on the route of transmission was only available for one patient, who contracted a wound infection after bathing in the Baltic Sea in October. However, no foreign travel was documented for these cases, so it can be assumed that these sporadic infections were acquired in Germany.

Infections spiked during summer heat waves in the Baltic Sea area.

Among patients who contracted wound infections, the majority (n = 32; 84%) had traumatic injuries either before or during contact with contaminated seawater. Another patient had superficial skin lesions as the result of mosquito bites, which served as an entry point for the *Vibrio* spp. In addition, five patients had underlying chronic skin diseases, including diabetic foot syndrome, lymphoedema, and atopic dermatitis, which predisposed them for wound infections.

Twenty-one patients with wound infections and one patient with gastroenteritis developed septicaemia, suggesting systemic disease progression. In addition, two patients developed pneumonia after seawater aspiration, and one patient presented with primary septicaemia without a known primary focus of infection. Wound infections and primary septicaemia mainly occurred in elderly patients (median age 69 and 78 years, respectively), and ear infections mainly occurred in younger patients (median age 13 years).

Information about the medical history and the course of disease was available for 65% (n = 41) of all patients. The most prevalent comorbidities were cardiovascular diseases (n = 25) and diabetes mellitus (n = 8). Eight patients were immunocompromised because of immunosuppressive therapy, underlying haematological malignancies, or splenectomy. The vast majority of patients had received antibiotic therapy (n = 37) and required hospitalisation (n = 35) with a mean time of hospitalisation of 18 days. Admission to an intensive care unit (ICU) was necessary for 21 patients. Eight patients died of the infection (Table 2).

**Table 2:** Deaths from *Vibrio* spp. infections, Germany, 2018 and 2019 (n = 8)

Year	Age group in years	*Vibrio* spp.	Clinical manifestation	Chronic disease	Immuno-suppression
2018	80−89	*V. vulnificus*	Wound infection	yes	yes
2018	70−79	*V. vulnificus*	Wound infection	yes	no
2018	70−79	*V. vulnificus*	Wound infection	yes	yes
2019	80−89	*V. cholerae* (non-O1/non-O139)	Gastroenteritis after eating crab meat	yes	yes
2019	80−89	*V. vulnificus*	Wound infection	yes	no
2019	60−69	*V. vulnificus*	Pneumogenic sepsis after drowning accident	no	no
2019	>90	*V. vulnificus*	Wound infection	yes	no
2019	50−59	*V. vulnificus*	Wound infection	yes	no

All but one of these eight patients were male, and the median age was 80 years (range: 56–93 years). Six patients died of wound infections from *V. vulnificus*, one patient died of gastroenteritis from *V. cholerae* (non-O1/non-O139) after eating crab meat, and one patient died of pneumonia from *V. vulnificus* after aspirating seawater. Of the patients who died of infection, three were immunocompromised and all but one had chronic diseases.

### Detected clinical *Vibrio* spp. isolates

Most infections were either attributed to *V. cholerae* (non–O1/non-O139) (n = 30; 48%) or *V. vulnificus* (n = 24; 38%) (Table 1). Less frequently, *V. parahaemolyticus* (n = 5; 8%), *V. fluvialis* (n = 3; 5%), and *V. alginolyticus* (n = 2; 3%) were detected.

Ear infections were primarily caused by *V. cholerae* (non–O1/non-O139) (n = 15/16), and wound infections were primarily caused by *V. vulnificus* (n = 22/38). One patient was infected with both *V. vulnificus* and *V. parahaemolyticus*; both species were detected in the wounds and in the blood cultures. One patient was diagnosed with polymicrobial ear infections with *V. parahaemolyticus* and *Shewanella putrefaciens*, and one patient was diagnosed with *V. cholerae*, *Staphylococcus aureus*, *Pseudomonas stutzeri*, and *E. coli*.

A total of 48 clinical *Vibrio* spp. isolates (*V. cholerae* (non–O1/non-O139), n = 29; *V. vulnificus*, n = 12; *V. parahaemolyticus*, n = 3; *V. alginolyticus*, n = 2; and *V. fluvialis*, n = 1) were available for further analysis and subsequently subjected to AST and WGS. One *V. vulnificus* isolate was only available for AST but not WGS, and one *V. vulnificus* isolate was only available for WGS but not for AST.

### Antimicrobial susceptibilities

Most of the 16 antibiotics tested in this study – e.g., tetracycline, third-generation cephalosporins (cefotaxime and ceftazidime), aminoglycosides (gentamicin and amikacin), and fluoroquinolones (ciprofloxacin and levofloxacin) – are recommended by the United States Centers for Disease Control and Prevention (CDC) for the treatment of *Vibrio* spp. infections [32]. Based on the AST results, the highest rate of phenotypic antimicrobial resistance of all *Vibrio* ssp. isolates in our study were detected for ampicillin (n = 6) and amikacin (n = 2). Low rates of resistance were found for gentamicin (n = 1) and amoxicillin-clavulanic acid (n = 1). However, all isolates were susceptible to cefepime, cefotaxime, ceftazidime, cefuroxime, chloramphenicol, ciprofloxacin, gentamicin, imipenem, meropenem, levofloxacin, and trimethoprim-sulfamethoxazole (Supplementary Table S1).

Resistance gene profiling identified resistance genes in 72% (34/47) of all *Vibrio* ssp. isolates (Table 3). In all but one ampicillin-resistant isolate, *bla_CARB_* genes encoding for a beta-lactamase were detected (*bla_CARB-7_*; n = 2). One of these isolates showed additional resistance to amoxicillin-clavulanic acid. Gentamicin-resistance is mostly conferred by aminoglycoside-modifying enzymes. However, no corresponding resistance gene was found for gentamicin-resistant and gentamicin-intermediate resistant isolates.

**Table 3:** Phenotypic antibiotic susceptibilities and associated genetic resistance markers in isolates from patients with *Vibrio* spp. infections, Germany, 2018 and 2019 (n = 48 isolates)

Antibiotic	Phenotypic susceptibility (n = 47)				Resistance detected by WGS (n = 47)		
	Resistant		Intermediate		Gene or mutation	Positive	
	n	%	n	%		n	%
AMK	2	4.3	11	23.4	NA	0	0
AMP	6	12.8	3	6.4	*bla_CARB-7_*	2	4.3
AMC	1	2.1	2	4.3	*bla_CARB-18_, bla_CARB-21_, bla_CARB-26_, bla_CARB-30_, bla_CARB-42_*	7	14.9
MEM	0	0	0	0	*varG*	15	31.9
TET	0	0	0	0	*tet* 34, *tet* 35	18	38.3
GEN	1	2.1	0	0	NA	0	0

AMC: amoxicillin-clavulanic acid; AMK: amikacin; AMP: ampicillin; GEN: gentamicin; MEM: meropenem; NA: not applicable; TET: tetracycline; WGS: whole genome sequencing.

Genomical analysis showed that *varG,* which encodes a metallo-beta-lactamase, was present in 31% (n = 15/29) of *V. cholerae* (non–O1/O139) isolates. VarG has beta-lactamase activity against penicillins, cephalosporins, and carbapenems, with the highest activity against meropenem [33], but no meropenem resistance was detected in our study isolates. Two *V. cholerae* (non–O1/non-O139) isolates additionally carried variants of *bla_CARB_*_–7_, a determinant of beta-lactam resistance.

The prevalence of antibiotic resistance genes *tet34* and *tet35* indicates intrinsic resistance to tetracycline, and these genes are present in all *V. vulnificus* and *V. parahaemolyticus* strains [34]. The function of *tet34* is associated with xanthine-guanine phosphoribosyltransferase (XPRT)-like activity and has been shown to be present in certain *Vibrio* spp [35]. However, the phenotype of antimicrobial resistance indicates that all strains are sensitive to tetracycline, suggesting that *tet34* is potentially non-functional [36].

### Phylogenetic data

**Figure 2:**
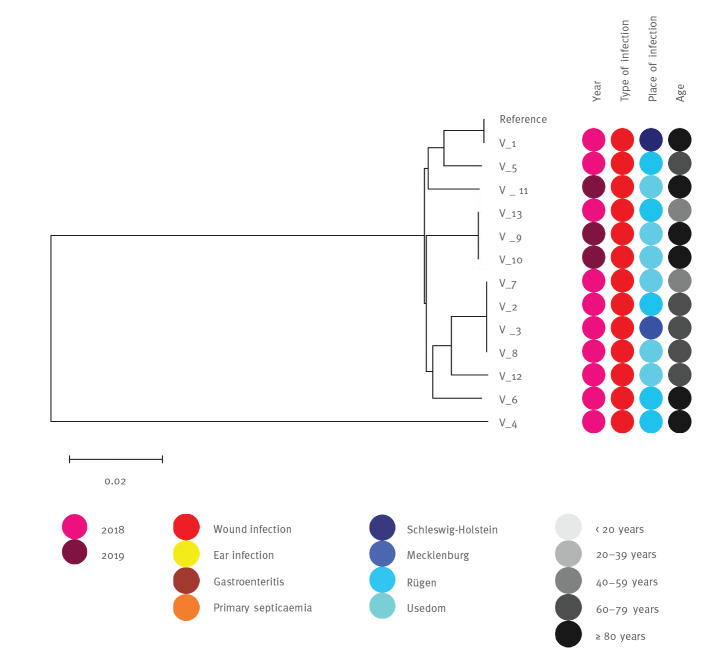
Maximum likelihood trees showing phylogenetic relationships of 13 *Vibrio vulnificus* genomes based on SNP analysis

**Figure 3:**
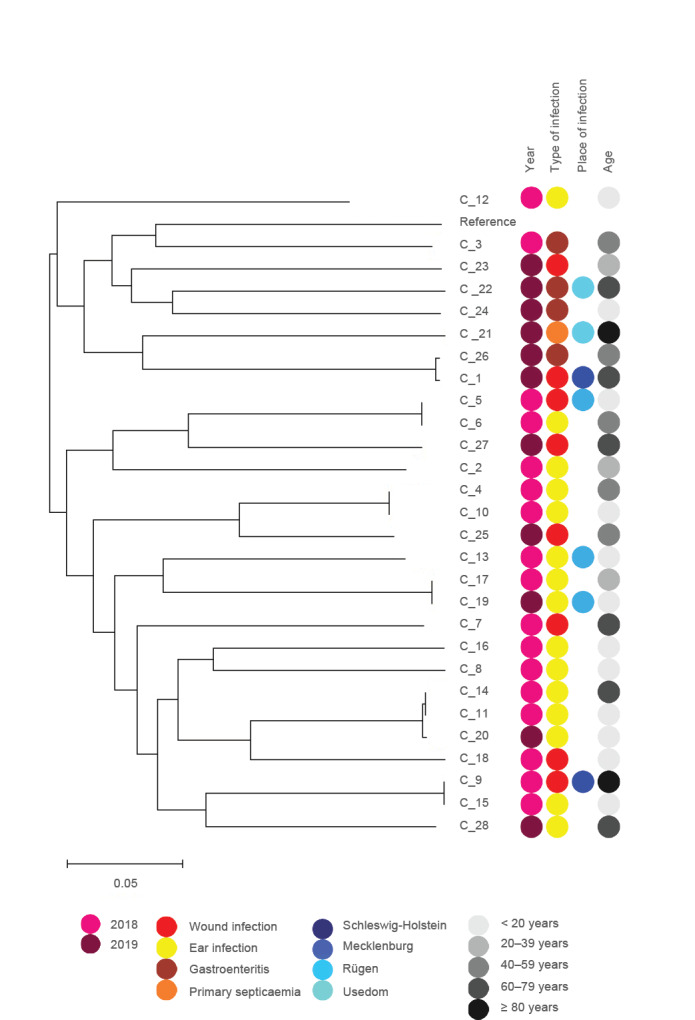
Maximum likelihood trees showing phylogenetic relationships of 28 *Vibrio cholerae* genomes based on SNP analysis

The genetic diversity of *V. vulnificus* isolates (n = 12) and *V. cholera* (non–O1/non-O139) isolates (n = 29) was evaluated using a phylogenomic tree based on single nucleotide polymorphisms (SNPs) (Figure 2 and 3). Despite the genomic divergence among clusters, no distinct patterns linking strain phylogeny, isolation source, or virulent capabilities were apparent. As the isolates within the same clade were closely related, we identified isolates that were identical in the detected SNPs, but mostly with different isolation sources. Virulence gene profiling did not reveal atypical virulence factors (e.g., toxins) in our study isolates (Supplementary Table S2).

SNP: single nucleotide polymorphism.

The *V. vulnificus* ASM221513v1 sequence was used as reference. Bar, 2% estimated sequence divergence.

SNP: single nucleotide polymorphism.

The *V. cholerae* ASM674v1 sequence was used as reference. Bar, 5% estimated sequence divergence.

### Climatological data

**Figure 4:**
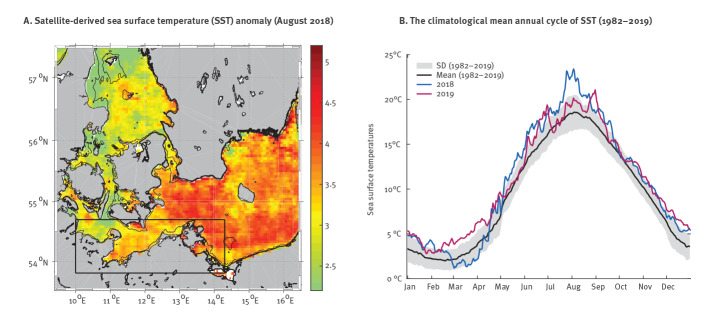
Sea surface temperatures in the south-eastern Baltic Sea in 2018 and 2019

Between 1982 and 2019, the SST warming trend in the south-western Baltic Sea (Box in Figure 4A) was 0.56 °C per decade. During this period, the Baltic Sea reached the highest SST in August 2018 (Figure 4A). The positive SST anomaly reached 2 °C throughout the south-western Baltic Sea. In the central-southern Baltic Sea and in some coastal areas, especially the eastern coasts with shallow areas, the positive SST anomaly even reached 5 °C. Although the SSTs in 2019 were not as high as in the summer of 2018, positive SST anomalies of up to 4 °C were observed in 2019, primarily in June and September (Figure 4B).

4A: Satellite-derived sea surface temperature (SST) anomaly in August 2018 with a reference to the climatological mean between 1982 and 2001. The thin black line shows the water depth of 10 m and reveals the shallow coastal areas. 4B: The climatological mean annual cycle of SST (black) based on data between 1982 and 2019 and averaged within the black rectangular box in 4A with the standard deviation underlaid as a grey shadow. The annual SSTs between 2018 and 2019 are shown in blue and pink as indicated in the legend.

## Discussion

Here, we present a large case series of domestically-acquired *Vibrio* infections in Germany. Consistent with previous studies [37,38], we demonstrate the broad clinical spectrum and various disease manifestations of *Vibrio* infections, ranging from mild gastroenteritis or ear infections to fulminant wound infections and primary septicaemia.

Also in line with previous findings, we observed a striking male predominance among patients with *Vibrio* infections [16,37]. While this may be attributed to sex differences in predisposing comorbidities and the choice of recreational activities, it has also been suggested that female sex hormones may have a protective effect against severe *V. vulnificus* infections [39]. Our study further emphasises that *Vibrio* infections occur across all age groups, but different disease manifestations have different age distributions. While ear infections most commonly affected children, wound infections and primary septicaemia were predominantly detected in elderly patients. The high prevalence of immunocompromising conditions and the chronic medical conditions among patients in our study cohort in general and in those who died of the infection in particular suggest these conditions may be determinants of disease severity in patients who contract *Vibrio* infections [37].

Importantly, recreational exposure to the Baltic Sea and its estuaries was responsible for the majority of infections in our study cohort. As in other studies, this finding demonstrates that most infections with *Vibrio* spp. involve either exposure of previously contracted wounds to seawater or injuries acquired during water-based activities [9,37]. The vast majority of *Vibrio* infections (n = 54; 86%) occurred between June and September: when water-associated recreational activities increase, high SSTs are responsible for an elevated abundance of *Vibrio* spp. in coastal waters. Sporadic infections outside these months were all caused by *V. cholerae* (non-O1/non-O139), which has a higher probability of occurrence at relatively low water temperatures compared to other *Vibrio* spp [4,40].

As the result of global warming, the SSTs in the Baltic Sea are expected to rise between 4 and 5 °C over the next several decades [41], which will likely lead to an increase of virulent *Vibrio* strains in coastal waters. On an interannual scale, the main driving force behind the Baltic Sea warming is the varying ambient temperature; less important are the intensity of solar radiation and the varying wind forces [42]. On a seasonal scale, the SSTs in the Baltic Sea are characterised by an average amplitude of 16 to 18 °C between summer and winter temperatures, which is higher in shallow bays and coastal regions as bathymetry prevents mixing and circulation of the water [43]. In addition, the rise of sea levels is expected to cause more coastal flooding, which will expand estuarine and brackish water environments suitable for *Vibrio* spp. growth [44]. Recently, early warning systems have been established that can forecast the environmental suitability of coastal waters for *Vibrio* spp. growth using remotely sensed SSTs and sea surface salinity measurements [40]. These systems can be used to alert healthcare providers and individuals at risk. Importantly, infection risk from *Vibrio* spp. does not correlate with faecal indicator organisms and therefore common water surveillance practices cannot predict infection risk from *Vibrio* spp [45]. To prevent severe infections, patients with open wounds or chronic skin diseases, especially those with underlying immunocompromising conditions, should be advised to avoid contact with seawater in the Baltic and Northern Sea area when water temperatures exceed 20 °C. In addition, vaccine candidates are being developed that may prevent *Vibrio* spp. infections in vulnerable populations [46].

Given the potentially fulminant disease course, patients with a presumptive diagnosis of severe *Vibrio*-associated diseases such as wound infections or primary septicaemia should promptly receive appropriate antibiotics for optimal disease outcomes. Recommended empiric treatment regimens include third-generation cephalosporins and either tetracyclines or fluoroquinolones [47]. In this study, the proportion of resistance among *Vibrio* spp. was comparable to a similar survey conducted on environmental isolates from marine coastal waters in Germany [22]. Using AST and WGS, we were able to show that empirical therapy would have been effective in all investigated cases. Resistance gene profiling identified some beta-lactamase and tetracycline inactivation enzyme genes, but no specific antimicrobial resistance patterns among *Vibrio* spp. isolates were detected that could pose a public health risk. Studies have shown that some strain subgroups are more likely to cause disease in humans than other subgroups, which is consistent with phylogenetic studies that show evidence of clustering of human isolates [48]. According to the SNP analysis, the strains showed three identical clusters for *V. vulnificus*, which caused wound infections. These clusters were found in three areas of the Baltic Sea coast, suggesting that clonal lines can spread across the Baltic Sea. However, because the number of reported *Vibrio*-associated diseases was relatively low, we could perform a phylogenetic analysis only between a small number of *V. vulnificus* and *V. cholerae* strains. Nevertheless, the results of the present survey provide an important resource for future prospective studies that focus on the emergence of *Vibrio* spp. infections contracted in the Baltic Sea and its estuaries.

Our study is subject to several limitations. First, it is not always possible to retrospectively differentiate bacterial colonisation from infection in patients with *Vibrio* spp. detected in wound or ear swabs after recent contact with seawater, especially in individuals with more than one potentially pathogenic agent. However, in patients with a typical clinical presentation and detection of human pathogenic *Vibrio* spp., the bacterium is highly likely the causative pathogen. Second, since non-cholera *Vibrio* spp. were not notifiable in Germany in 2018 and 2019, we aimed to contact all hospitals and microbiological laboratories near the German Baltic Sea and Northern Sea; however, this data collection approach meant that *Vibrio* infections detected elsewhere were probably not captured. In addition, patients with self-limiting and transient infections may not have undergone microbiological testing or sought medical attention. Although this selection bias may result in overestimation of disease severity in our study cohort, the actual clinical burden associated with *Vibrio* infections may be grossly underestimated due to under-reporting and underdiagnosis. Third, we were not able to obtain reliable information on comorbidities, disease course, and place of infection from all patients. These limitations underscore the need for mandatory standardised surveillance systems for *Vibrio* spp. infections [2,6,49]. In Germany, a mandatory notification for *Vibrio* spp. infections has been in place since March 2020. In 2020, only 13 *Vibrio* spp. infections were reported to the RKI. This relatively low number of cases was probably the result of restrictions imposed during the coronavirus disease (COVID-19) pandemic. In the future, this mandatory surveillance may help capture comprehensive clinical and microbiological data. These data are paramount for obtaining the precise epidemiological and clinical impact of domestically-acquired *Vibrio* infections, identifying vulnerable populations, and guiding future public health preparedness activities.

### Conclusion

Our study suggests that severe heatwaves during the summer of 2018 and 2019 were responsible for at least 63 *Vibrio* spp. infections contracted in the German Baltic Sea and its estuaries. Since global warming is predicted to favour the proliferation of these bacteria in the aquatic environment and demographic change will likely increase the number of vulnerable individuals, a rise in severe *Vibrio*-associated diseases may be expected in the future. Although the burden of disease from *Vibrio* spp. infections is currently relatively low, the potentially fulminant disease course in patients with wound infections and primary septicaemia underlines the need to protect vulnerable population groups.

## German *Vibrio* Study Group

Veronika Balau (MVZ IMD Labor Greifswald GmbH, Greifswald, Germany); Elsa Baufeld (Friedrich Loeffler-Institute of Medical Microbiology, University Hospital Greifswald, Germany); Simone Brechmann (MVZ Diamedis, Bielefeld, Germany); Lutz Briedigkeit (Institute for Laboratory and Transfusion Medicine, Helios Kliniken Schwerin, Schwerin, Germany); Stephan Diedrich (Department of General, Visceral, Thoracic and Vascular Surgery, Universitätsmedizin Greifswald, Greifswald, Germany); Ulrike Ebert (Kreiskrankenhaus Frankenberg, Frankenberg (Eder), Germany); Helmut Fickenscher (Institute for Infection Medicine, University of Kiel and University Hospital Schleswig-Holstein, Kiel, Germany); Bojana Grgic (Department of Hygiene and Infection Prevention, Helios Kliniken Schwerin, Schwerin, Germany); Claus-Dieter Heidecke (Department of General, Visceral, Thoracic and Vascular Surgery, University Medicine of Greifswald, Greifswald, Germany); Peter Hinz (Department of Trauma and Orthopaedic Surgery, Clinic of Surgery, University Medicine Greifswald, Greifswald, Germany); Ada Hoffmann (Department of Infectious Diseases and Microbiology, University Hospital Schleswig-Holstein, Lübeck, Germany); Meike Holbe (Department of Paediatrics, Medical University of Lübeck, Lübeck, Germany); Ralf Ignatius (MVZ Labor 28 GmbH, Berlin, Germany, and Charité, Institute of Microbiology, Infectious Diseases and Immunology, Berlin, Germany); Olaf Kaup (Microbiologic Laboratory, Klinikum Bielefeld Rosenhöhe, Bielefeld, Germany); Martin Kern (IMD Labor Oderland GmbH, Frankfurt (Oder), Germany); Martina Kerwat (Institute of Medical Microbiology and Hygiene, Philipps University Marburg, Marburg, Germany); Ingo Klempien (Department of Clinical Hygiene and Infectious Diseases, Helios Hanseklinikum Stralsund, Stralsund, Germany); Georg Lamprecht (Department of Medicine II, Division of Gastroenterology and Endocrinology, University Medical Center Rostock, Rostock, Germany); Astrid Meerbach (MVZ Labor Dr Reising-Ackermann und Kollegen, Leipzig, Germany); Alexander Mischnik (Department of Infectious Diseases and Microbiology, University of Lübeck, Lübeck, Germany); Andreas Podbielski (Institute of Medical Microbiology, Virology and Hygiene, University Hospital Rostock, Rostock, Germany); Stephan Schaefer (MVZ Labor Limbach Vorpommern-Rügen GmbH, Stralsund, Germany); Roman Schwarz (Labor Mönchengladbach, MVZ Dr. Stein + Kollegen, Mönchengladbach, Germany); Eckhard Strauch (Bundesinstitut für Risikobewertung (BfR) - German Federal Institute for Risk Assessment, Berlin, Germany; Philipp Warnke (Institute of Medical Microbiology, Virology and Hygiene, University Hospital Rostock, Rostock, Germany); Simone Weikert-Asbeck (Department of Infectious Diseases and Microbiology, University of Lübeck, Lübeck, Germany); Maria Witte (Department of General, Thoracic, Vascular, and Transplantation Surgery, Rostock University Medical Center, Rostock, Germany); Waleed Zaki (Berlin, Germany).

## Supplementary Data

510000 Supplementary Material
