# Supplementary material for: Heatwave-associated Vibrio infections in Germany, 2018 and 2019
Source: Euro Surveill. 2021 Oct 14;26(41):2002041. doi: 10.2807/1560-7917.ES.2021.26.41.2002041 (PMC8518310; doi:10.2807/1560-7917.ES.2021.26.41.2002041)
Supplement: Supplementary Material [file 20-02041_BREHM_Supplementary_material.pdf]

This supplementary material is hosted by *Eurosurveillance* as supporting information alongside the article “Heatwave-associated Vibrio infections in Germany, 2018 and 2019“, on behalf of the authors, who remain responsible for the accuracy and appropriateness of the content. The same standards for ethics, copyright, attributions and permissions as for the article apply. Supplements are not edited by *Eurosurveillance* and the journal is not responsible for the maintenance of any links or email addresses provided therein.

Supplementary Table 1 – Antimicrobial susceptibilities and resistance genes of all clinical *Vibrio* spp. isolates

Isolate	*Vibrio* spp.	AMK	AMC	AMP	FEP	CTX	CAZ	CXM	CHL	CIP	GEN	IPM	LVX	MEM	TZP	SXT	TET	AZM	DOX	Resistance genes
C_1	*V. cholerae*	I	S	S	S	S	S	S	S	S	S	S	S	S	S	S	S	S	S	
C_2	*V. cholerae*	S	S	S	S	S	S	S	S	S	S	S	S	S	S	S	S	S	S	
C_3	*V. cholerae*	S	S	S	S	S	S	S	S	S	S	S	S	S	S	S	S	S	S	*varG*
C_4	*V. cholerae*	S	S	S	S	S	S	S	S	S	S	S	S	S	S	S	S	S	S	*varG*
C_5	*V. cholerae*	S	I	R	S	S	S	S	S	S	S	S	S	S	S	S	S	S	S	
C_6	*V. cholerae*	S	S	S	S	S	S	S	S	S	S	S	S	S	S	S	S	S	S	
C_7	*V. cholerae*	S	S	S	S	S	S	S	S	S	S	S	S	S	S	S	S	S	S	
C_8	*V. cholerae*	S	S	S	S	S	S	S	S	S	S	S	S	S	S	S	S	S	S	*varG*
C_9	*V. cholerae*	S	S	S	S	S	S	S	S	S	S	S	S	S	S	S	S	S	S	*varG*
C_10	*V. cholerae*	S	S	S	S	S	S	S	S	S	S	S	S	S	S	S	S	S	S	*varG*
C_11	*V. cholerae*	S	S	S	S	S	S	S	S	S	S	S	S	S	S	S	S	S	S	*varG*
C_12	*V. cholerae*	S	S	S	S	S	S	S	S	S	S	S	S	S	S	S	S	S	S	
C_13	*V. cholerae*	S	S	S	S	S	S	S	S	S	S	S	S	S	S	S	S	S	S	
C_14	*V. cholerae*	S	S	S	S	S	S	S	S	S	S	S	S	S	S	S	S	S	S	*varG*
C_15	*V. cholerae*	S	S	S	S	S	S	S	S	S	S	S	S	S	S	S	S	S	S	*varG*
C_16	*V. cholerae*	S	S	S	S	S	S	S	S	S	S	S	S	S	S	S	S	S	S	*varG*
C_17	*V. cholerae*	S	S	S	S	S	S	S	S	S	S	S	S	S	S	S	S	S	S	
C_18	*V. cholerae*	S	S	R	S	S	S	S	S	S	S	S	S	S	S	S	S	S	S	*blaCarb-7; varG*
C_19	*V. cholerae*	S	S	S	S	S	S	S	S	S	S	S	S	S	S	S	S	S	S	
C_20	*V. cholerae*	S	S	S	S	S	S	S	S	S	S	S	S	S	S	S	S	S	S	*varG*
C_21	*V. cholerae*	S	S	S	S	S	S	S	S	S	S	S	S	S	S	S	S	S	S	*varG*
C_22	*V. cholerae*	S	S	S	S	S	S	S	S	S	S	S	S	S	S	S	S	S	S	*varG*
C_23	*V. cholerae*	S	S	S	S	S	S	S	S	S	S	S	S	S	S	S	S	S	S	
C_24	*V. cholerae*	S	S	S	S	S	S	S	S	S	S	S	S	S	S	S	S	S	S	*varG*
C_25	*V. cholerae*	S	S	S	S	S	S	S	S	S	S	S	S	S	S	S	S	S	S	*varG*
C_26	*V. cholerae*	S	S	S	S	S	S	S	S	S	S	S	S	S	S	S	S	S	S	
C_27	*V. cholerae*	S	I	R	S	S	S	S	S	S	S	S	S	S	S	S	S	S	S	*blaCarb-7*
C_28	*V. cholerae*	S	S	S	S	S	S	S	S	S	S	S	S	S	S	S	S	S	S	
V_1	*V. vulnificus*	I	S	S	S	S	S	S	S	S	I	S	S	S	S	S	S	S	S	*tet(34)*
V_2	*V. vulnificus*	S	S	S	S	S	S	S	S	S	S	S	S	S	S	S	S	S	S	*tet(34)*
V_4	*V. vulnificus*	I	S	S	S	S	S	S	S	S	S	S	S	S	S	S	S	S	S	*tet(34)*
V_5	*V. vulnificus*	I	S	S	S	S	S	S	S	S	I	S	S	S	S	S	S	S	S	*tet(34)*
V_6	*V. vulnificus*	S	S	S	S	S	S	S	S	S	S	S	S	S	S	S	S	S	S	*tet(34)*
V_7	*V. vulnificus*	I	S	S	S	S	S	S	S	S	S	S	S	S	S	S	S	S	S	*tet(34)*
V_8	*V. vulnificus*	S	S	S	S	S	S	S	S	S	S	S	S	S	S	S	S	S	S	*tet(34)*
V_9	*V. vulnificus*	R	S	S	S	S	S	S	S	S	I	S	S	S	S	S	S	S	S	*tet(34)*
V_10	*V. vulnificus*	R	S	S	S	S	S	S	S	S	I	S	S	S	S	S	S	S	S	*tet(34)*
V_11	*V. vulnificus*	I	S	S	S	S	S	S	S	S	I	S	S	S	S	S	S	S	S	*tet(34)*
V_12	*V. vulnificus*	I	S	S	S	S	S	S	S	S	S	S	S	S	S	S	S	S	S	*tet(34)*
V_13	*V. vulnificus*	I	S	S	S	S	S	S	S	S	I	S	S	S	S	S	S	S	S	*tet(34)*
V_14	*V. vulnificus*	I	S	S	S	S	S	S	S	S	S	S	S	S	S	S	S	S	S	*tet(34)*
A_1	*V. alginolyticus*	S	S	R	S	S	S	S	S	S	I	S	S	S	S	S	S	S	S	*blaCarb-42; tet(34); tet(35)*
A_2	*V. alginolyticus*	I	S	R	S	S	S	S	S	S	I	S	S	S	S	S	S	S	S	*blaCarb-42; tet(34); tet(35)*
P_1	*V. parahaemolyticus*	S	S	I	S	S	S	S	S	S	S	S	S	S	S	S	S	S	S	*blaCarb-21; blaCarb-30; tet(34); tet(35)*
P_2	*V. parahaemolyticus*	S	S	I	S	S	S	S	S	S	R	S	S	S	S	S	S	S	S	*blaCarb-26; tet(34); tet(35)*
P_3	*V. parahaemolyticus*	I	S	I	S	S	S	S	S	S	S	S	S	S	S	S	S	S	S	*blaCarb-26; blaCarb-18; tet(34); tet(35)*
F_1	*V. fluvialis*	S	S	S	S	S	S	S	S	S	S	S	S	S	S	S	S	S	S	
F_2	*V. fluvialis*	S	R	R	S	S	S	R	S	S	S	S	S	S	S	S	S	S	S	

AMK, amikacin; AMC, amoxicillin-clavulanic acid; AMP, ampicillin; FEP, cefepime; CTX, ceftriaxone; CAZ, ceftazidime; CXM, cefuroxime; CHL, chloramphenicol; CIP, ciprofloxacin; GEN, gentamicin; IPM, imipenem; LVX, levofloxacin; MEM, meropenem; TZP, piperacillin-tazobactam; SXT, trimethoprim-sulfamethoxazole; TET, tetracycline; AZM, azithromycin; DOX, doxycycline

Supplemental Table 2 – Virulence gene profiling of all clinical *Vibrio* spp. isolates

Isolate	number	genes
A_1	14	cheY,flgB,tlh,tyeA,vcrH,vopB,vopD,vopR,vscF,vscI,vscN,vscR,vscS,vscU
A_2	14	cheY,flgB,tlh,tyeA,vcrH,vopB,vopD,vopR,vscF,vscI,vscN,vscR,vscS,vxsC
C_1	110	VCA0109,VCA0122,VPA1340,VPA1353,cheA,cheB,cheR,cheV,cheW,cheY,cheZ,clpB/vasG,cqsA,flaA,flaB,flaC,flaD,flaE,flaG,flaI,fleN,fleR/flrC,fleS/flrB,flgA,flgB,flgC,flgD,flgE,flgF,flgG,flgH,flgI,flgJ,flgK,flgL,flgM,flgN,flgO,flgP,flgT,flhA,flhB,flhF,fliA,fliD,fliE,fliF,fliG,fliH,fliI,fliJ,fliK,fliL,fliM,fliN,fliO,fliP,fliQ,fliR,fliS,flrA,hcp-2,hlyA,icmF/vasK,luxS,motA,motB,motX,motY,mshB,mshC,mshD,mshE,mshF,mshG,mshH,mshI,mshJ,mshK,mshL,mshM,mshN,rtxA,rtxB,rtxC,rtxD,vasA,vasB,vasC,vasD,vasE,vasF,vasH,vasI,vasJ,vasL,vcrD2,vgrG-2,vipA/mglA,vipB/mglB,vopB2,vopD2,vscC2,vscJ2,vscN2,vscQ2,vscR2,vscS2,vscT2,vscU2,
C_2	79	cheA,cheB,cheR,cheV,cheW,cheY,cheZ,flaA,flaB,flaC,flaD,flaE,flaG,flaI,fleN,fleR/flrC,fleS/flrB,flgA,flgB,flgC,flgD,flgE,flgF,flgG,flgH,flgI,flgJ,flgK,flgL,flgM,flgN,flgO,flgP,flgT,flhA,flhB,flhF,fliA,fliD,fliE,fliF,fliG,fliH,fliI,fliJ,fliK,fliL,fliM,fliN,fliO,fliP,fliQ,fliR,fliS,flrA,hcp-2,hlyA,luxS,motA,motB,motX,motY,mshB,mshC,mshD,mshE,mshF,mshG,mshH,mshI,mshJ,mshK,mshL,mshM,mshN,rtxB,rtxC,rtxD,vgrG-2
C_3	96	VCA0109,VCA0122,cheA,cheB,cheR,cheV,cheW,cheY,cheZ,clpB/vasG,cqsA,flaA,flaB,flaC,flaD,flaE,flaG,flaI,fleN,fleR/flrC,fleS/flrB,flgA,flgB,flgC,flgD,flgE,flgF,flgG,flgH,flgI,flgJ,flgK,flgL,flgM,flgN,flgO,flgP,flgT,flhA,flhB,flhF,fliA,fliD,fliE,fliF,fliG,fliH,fliI,fliJ,fliK,fliL,fliM,fliN,fliO,fliP,fliQ,fliR,fliS,flrA,hcp-2,hlyA,icmF/vasK,luxS,motA,motB,motX,motY,mshA,mshB,mshC,mshD,mshE,mshF,mshG,mshH,mshI,mshJ,mshK,mshL,mshM,mshN,rtxB,rtxC,rtxD,vasA,vasB,vasC,vasD,vasE,vasF,vasH,vasI,vasJ,vgrG-2,vipA/mglA,vipB/mglB,
C_4	96	VCA0109,VCA0122,cheA,cheB,cheR,cheV,cheW,cheY,cheZ,clpB/vasG,cqsA,flaA,flaB,flaC,flaD,flaE,flaG,flaI,fleN,fleR/flrC,fleS/flrB,flgA,flgB,flgC,flgD,flgE,flgF,flgG,flgH,flgI,flgJ,flgK,flgL,flgM,flgN,flgO,flgP,flgT,flhA,flhB,flhF,fliA,fliD,fliE,fliF,fliG,fliH,fliI,fliJ,fliK,fliL,fliM,fliN,fliO,fliP,fliQ,fliR,fliS,flrA,hcp-2,hlyA,icmF/vasK,luxS,motA,motB,motX,motY,mshB,mshC,mshD,mshE,mshF,mshG,mshH,mshI,mshJ,mshK,mshL,mshM,mshN,rtxB,rtxC,rtxD,vasA,vasB,vasC,vasD,vasE,vasF,vasH,vasI,vasJ,vasL,vgrG-2,vipA/mglA,vipB/mglB,
C_5	95	VCA0109,VCA0122,cheA,cheB,cheR,cheV,cheW,cheY,cheZ,clpB/vasG,flaA,flaB,flaC,flaD,flaE,flaG,flaI,fleN,fleR/flrC,fleS/flrB,flgA,flgB,flgC,flgD,flgE,flgF,flgG,flgH,flgI,flgJ,flgK,flgL,flgM,flgN,flgO,flgP,flgT,flhA,flhB,flhF,fliA,fliD,fliE,fliF,fliG,fliH,fliI,fliJ,fliK,fliL,fliM,fliN,fliO,fliP,fliQ,fliR,fliS,flrA,hcp-2,hlyA,icmF/vasK,luxS,motA,motB,motX,motY,mshB,mshC,mshD,mshE,mshF,mshG,mshH,mshI,mshJ,mshK,mshL,mshM,mshN,rtxB,rtxC,rtxD,vasA,vasB,vasC,vasD,vasE,vasF,vasH,vasI,vasJ,vasL,vgrG-3,vipA/mglA,vipB/mglB,
C_6	95	VCA0109,VCA0122,cheA,cheB,cheR,cheV,cheW,cheY,cheZ,clpB/vasG,flaA,flaB,flaC,flaD,flaE,flaG,flaI,fleN,fleR/flrC,fleS/flrB,flgA,flgB,flgC,flgD,flgE,flgF,flgG,flgH,flgI,flgJ,flgK,flgL,flgM,flgN,flgO,flgP,flgT,flhA,flhB,flhF,fliA,fliD,fliE,fliF,fliG,fliH,fliI,fliJ,fliK,fliL,fliM,fliN,fliO,fliP,fliQ,fliR,fliS,flrA,hcp-2,hlyA,icmF/vasK,luxS,motA,motB,motX,motY,mshB,mshC,mshD,mshE,mshF,mshG,mshH,mshI,mshJ,mshK,mshL,mshM,mshN,rtxB,rtxC,rtxD,vasA,vasB,vasC,vasD,vasE,vasF,vasH,vasI,vasJ,vasL,vgrG-3,vipA/mglA,vipB/mglB,
C_7	96	VCA0109,VCA0122,cheA,cheB,cheR,cheV,cheW,cheY,cheZ,clpB/vasG,cqsA,flaA,flaB,flaC,flaD,flaE,flaG,flaI,fleN,fleR/flrC,fleS/flrB,flgA,flgB,flgC,flgD,flgE,flgF,flgG,flgH,flgI,flgJ,flgK,flgL,flgM,flgN,flgO,flgP,flgT,flhA,flhB,flhF,fliA,fliD,fliE,fliF,fliG,fliH,fliI,fliJ,fliK,fliL,fliM,fliN,fliO,fliP,fliQ,fliR,fliS,flrA,hcp-2,hlyA,icmF/vasK,luxS,motA,motB,motX,motY,mshB,mshC,mshD,mshE,mshF,mshG,mshH,mshI,mshJ,mshK,mshL,mshM,mshN,rtxB,rtxC,rtxD,vasA,vasB,vasC,vasD,vasE,vasF,vasH,vasI,vasJ,vasL,vgrG-2,vipA/mglA,vipB/mglB,
C_8	97	VCA0109,VCA0122,cheA,cheB,cheR,cheV,cheW,cheY,cheZ,clpB/vasG,cqsA,flaA,flaB,flaC,flaD,flaE,flaG,flaI,fleN,fleR/flrC,fleS/flrB,flgA,flgB,flgC,flgD,flgE,flgF,flgG,flgH,flgI,flgJ,flgK,flgL,flgM,flgN,flgO,flgP,flgT,flhA,flhB,flhF,fliA,fliD,fliE,fliF,fliG,fliH,fliI,fliJ,fliK,fliL,fliM,fliN,fliO,fliP,fliQ,fliR,fliS,flrA,hcp-2,hlyA,icmF/vasK,luxS,motA,motB,motX,motY,mshB,mshC,mshD,mshE,mshF,mshG,mshH,mshI,mshJ,mshK,mshL,mshM,mshN,rtxB,rtxC,rtxD,vasA,vasB,vasC,vasD,vasE,vasF,vasH,vasI,vasJ,vasL,vgrG-2,vgrG-3,vipA/mglA,vipB/mglB,
C_9	95	VCA0109,VCA0122,cheA,cheB,cheR,cheV,cheW,cheY,cheZ,clpB/vasG,cqsA,flaA,flaB,flaC,flaD,flaE,flaG,flaI,fleN,fleR/flrC,fleS/flrB,flgA,flgB,flgC,flgD,flgE,flgF,flgG,flgH,flgI,flgJ,flgK,flgL,flgM,flgN,flgO,flgP,flgT,flhA,flhB,flhF,fliA,fliD,fliE,fliF,fliG,fliH,fliI,fliJ,fliK,fliL,fliM,fliN,fliO,fliP,fliQ,fliR,fliS,flrA,hcp-2,hlyA,icmF/vasK,luxS,motA,motB,motX,motY,mshB,mshC,mshD,mshE,mshF,mshG,mshH,mshI,mshJ,mshK,mshL,mshM,mshN,rtxB,rtxC,rtxD,vasA,vasB,vasC,vasD,vasE,vasF,vasH,vasI,vasJ,vgrG-3,vipA/mglA,vipB/mglB,
C_10	96	VCA0109,VCA0122,cheA,cheB,cheR,cheV,cheW,cheY,cheZ,clpB/vasG,cqsA,flaA,flaB,flaC,flaD,flaE,flaG,flaI,fleN,fleR/flrC,fleS/flrB,flgA,flgB,flgC,flgD,flgE,flgF,flgG,flgH,flgI,flgJ,flgK,flgL,flgM,flgN,flgO,flgP,flgT,flhA,flhB,flhF,fliA,fliD,fliE,fliF,fliG,fliH,fliI,fliJ,fliK,fliL,fliM,fliN,fliO,fliP,fliQ,fliR,fliS,flrA,hcp-2,hlyA,icmF/vasK,luxS,motA,motB,motX,motY,mshB,mshC,mshD,mshE,mshF,mshG,mshH,mshI,mshJ,mshK,mshL,mshM,mshN,rtxB,rtxC,rtxD,vasA,vasB,vasC,vasD,vasE,vasF,vasH,vasI,vasJ,vasL,vgrG-2,vipA/mglA,vipB/mglB,

C_11	95	VCA0109,VCA0122,cheA,cheB,cheR,cheV,cheW,cheY,cheZ,clpB/vasG,cqsA,flaA,flaB,flaC,flaD,flaE,flaG,flaI,fleN,fleR/flrC,fleS/flrB,flgA,flgB,flgC,flgD,flgE,flgF,flgG,flgH,flgI,flgJ,flgK,flgL,flgM,flgN,flgO,flgP,flgT,flhA,flhB,flhF,fliA,fliD,fliE,fliF,fliG,fliH,fliI,fliJ,fliK,fliL,fliM,fliN,fliO,fliP,fliQ,fliR,fliS,flrA,hcp-2,hlyA,icmF/vasK,luxS,motA,motB,motX,motY,mshB,mshC,mshD,mshE,mshF,mshG,mshH,mshI,mshJ,mshK,mshL,mshM,mshN,rtxB,rtxC,rtxD,vasA,vasB,vasC,vasD,vasE,vasF,vasH,vasI,vasJ,vasL,vipA/mglA,vipB/mglB,
C_12	95	VCA0109,VCA0122,cheA,cheB,cheR,cheV,cheW,cheY,cheZ,clpB/vasG,cqsA,flaA,flaB,flaC,flaD,flaE,flaG,flaI,fleN,fleR/flrC,fleS/flrB,flgA,flgB,flgC,flgD,flgE,flgF,flgG,flgH,flgI,flgJ,flgK,flgL,flgM,flgN,flgO,flgP,flgT,flhA,flhB,flhF,fliA,fliD,fliE,fliF,fliG,fliH,fliI,fliJ,fliK,fliL,fliM,fliN,fliO,fliP,fliQ,fliR,fliS,flrA,hcp-2,hlyA,icmF/vasK,luxS,motA,motB,motX,motY,mshB,mshC,mshD,mshE,mshF,mshG,mshH,mshI,mshJ,mshK,mshL,mshM,mshN,rtxB,rtxC,rtxD,vasA,vasB,vasC,vasD,vasE,vasF,vasH,vasI,vasJ,vasL,vipA/mglA,vipB/mglB,
C_13	96	VCA0109,VCA0122,cheA,cheB,cheR,cheV,cheW,cheY,cheZ,clpB/vasG,cqsA,flaA,flaB,flaC,flaD,flaE,flaG,flaI,fleN,fleR/flrC,fleS/flrB,flgA,flgB,flgC,flgD,flgE,flgF,flgG,flgH,flgI,flgJ,flgK,flgL,flgM,flgN,flgO,flgP,flgT,flhA,flhB,flhF,fliA,fliD,fliE,fliF,fliG,fliH,fliI,fliJ,fliK,fliL,fliM,fliN,fliO,fliP,fliQ,fliR,fliS,flrA,hcp-2,hlyA,icmF/vasK,luxS,motA,motB,motX,motY,mshB,mshC,mshD,mshE,mshF,mshG,mshH,mshI,mshJ,mshK,mshL,mshM,mshN,rtxB,rtxC,rtxD,vasA,vasB,vasC,vasD,vasE,vasF,vasH,vasI,vasJ,vasL,vgrG-2,vipA/mglA,vipB/mglB,
C_14	95	VCA0109,VCA0122,cheA,cheB,cheR,cheV,cheW,cheY,cheZ,clpB/vasG,cqsA,flaA,flaB,flaC,flaD,flaE,flaG,flaI,fleN,fleR/flrC,fleS/flrB,flgA,flgB,flgC,flgD,flgE,flgF,flgG,flgH,flgI,flgJ,flgK,flgL,flgM,flgN,flgO,flgP,flgT,flhA,flhB,flhF,fliA,fliD,fliE,fliF,fliG,fliH,fliI,fliJ,fliK,fliL,fliM,fliN,fliO,fliP,fliQ,fliR,fliS,flrA,hcp-2,hlyA,icmF/vasK,luxS,motA,motB,motX,motY,mshB,mshC,mshD,mshE,mshF,mshG,mshH,mshI,mshJ,mshK,mshL,mshM,mshN,rtxB,rtxC,rtxD,vasA,vasB,vasC,vasD,vasE,vasF,vasH,vasI,vasJ,vasL,vipA/mglA,vipB/mglB,
C_15	96	VCA0109,VCA0122,cheA,cheB,cheR,cheV,cheW,cheY,cheZ,clpB/vasG,cqsA,flaA,flaB,flaC,flaD,flaE,flaG,flaI,fleN,fleR/flrC,fleS/flrB,flgA,flgB,flgC,flgD,flgE,flgF,flgG,flgH,flgI,flgJ,flgK,flgL,flgM,flgN,flgO,flgP,flgT,flhA,flhB,flhF,fliA,fliD,fliE,fliF,fliG,fliH,fliI,fliJ,fliK,fliL,fliM,fliN,fliO,fliP,fliQ,fliR,fliS,flrA,hcp-2,hlyA,icmF/vasK,luxS,motA,motB,motX,motY,mshB,mshC,mshD,mshE,mshF,mshG,mshH,mshI,mshJ,mshK,mshL,mshM,mshN,rtxB,rtxC,rtxD,vasA,vasB,vasC,vasD,vasE,vasF,vasH,vasI,vasJ,vasL,vgrG-3,vipA/mglA,vipB/mglB,
C_16	96	VCA0109,VCA0122,cheA,cheB,cheR,cheV,cheW,cheY,cheZ,clpB/vasG,cqsA,flaA,flaB,flaC,flaD,flaE,flaG,flaI,fleN,fleR/flrC,fleS/flrB,flgA,flgB,flgC,flgD,flgE,flgF,flgG,flgH,flgI,flgJ,flgK,flgL,flgM,flgN,flgO,flgP,flgT,flhA,flhB,flhF,fliA,fliD,fliE,fliF,fliG,fliH,fliI,fliJ,fliK,fliL,fliM,fliN,fliO,fliP,fliQ,fliR,fliS,flrA,hcp-2,hlyA,icmF/vasK,luxS,motA,motB,motX,motY,mshB,mshC,mshD,mshE,mshF,mshG,mshH,mshI,mshJ,mshK,mshL,mshM,mshN,rtxB,rtxC,rtxD,vasA,vasB,vasC,vasD,vasE,vasF,vasH,vasI,vasJ,vasL,vgrG-2,vipA/mglA,vipB/mglB,
C_17	98	VCA0109,VCA0122,cheA,cheB,cheR,cheV,cheW,cheY,cheZ,clpB/vasG,cqsA,flaA,flaB,flaC,flaD,flaE,flaG,flaI,fleN,fleR/flrC,fleS/flrB,flgA,flgB,flgC,flgD,flgE,flgF,flgG,flgH,flgI,flgJ,flgK,flgL,flgM,flgN,flgO,flgP,flgT,flhA,flhB,flhF,fliA,fliD,fliE,fliF,fliG,fliH,fliI,fliJ,fliK,fliL,fliM,fliN,fliO,fliP,fliQ,fliR,fliS,flrA,hcp-2,hlyA,icmF/vasK,luxS,motA,motB,motX,motY,mshB,mshC,mshD,mshE,mshF,mshG,mshH,mshI,mshJ,mshK,mshL,mshM,mshN,rtxA,rtxB,rtxC,rtxD,vasA,vasB,vasC,vasD,vasE,vasF,vasH,vasI,vasJ,vasL,vgrG-2,vgrG-3,vipA/mglA,vipB/mglB
C_18	96	VCA0109,VCA0122,cheA,cheB,cheR,cheV,cheW,cheY,cheZ,clpB/vasG,cqsA,flaA,flaB,flaC,flaD,flaE,flaG,flaI,fleN,fleR/flrC,fleS/flrB,flgA,flgB,flgC,flgD,flgE,flgF,flgG,flgH,flgI,flgJ,flgK,flgL,flgM,flgN,flgO,flgP,flgT,flhA,flhB,flhF,fliA,fliD,fliE,fliF,fliG,fliH,fliI,fliJ,fliK,fliL,fliM,fliN,fliO,fliP,fliQ,fliR,fliS,flrA,hcp-2,hlyA,icmF/vasK,luxS,motA,motB,motX,motY,mshB,mshC,mshD,mshE,mshF,mshG,mshH,mshI,mshJ,mshK,mshL,mshM,mshN,rtxB,rtxC,rtxD,vasA,vasB,vasC,vasD,vasE,vasF,vasH,vasI,vasJ,vasL,vgrG-2,vipA/mglA,vipB/mglB
C_19	98	VCA0109,VCA0122,cheA,cheB,cheR,cheV,cheW,cheY,cheZ,clpB/vasG,cqsA,flaA,flaB,flaC,flaD,flaE,flaG,flaI,fleN,fleR/flrC,fleS/flrB,flgA,flgB,flgC,flgD,flgE,flgF,flgG,flgH,flgI,flgJ,flgK,flgL,flgM,flgN,flgO,flgP,flgT,flhA,flhB,flhF,fliA,fliD,fliE,fliF,fliG,fliH,fliI,fliJ,fliK,fliL,fliM,fliN,fliO,fliP,fliQ,fliR,fliS,flrA,hcp-2,hlyA,icmF/vasK,luxS,motA,motB,motX,motY,mshB,mshC,mshD,mshE,mshF,mshG,mshH,mshI,mshJ,mshK,mshL,mshM,mshN,rtxA,rtxB,rtxC,rtxD,vasA,vasB,vasC,vasD,vasE,vasF,vasH,vasI,vasJ,vasL,vgrG-2,vgrG-3,vipA/mglA,vipB/mglB,
C_20	96	VCA0109,VCA0122,cheA,cheB,cheR,cheV,cheW,cheY,cheZ,clpB/vasG,cqsA,flaA,flaB,flaC,flaD,flaE,flaG,flaI,fleN,fleR/flrC,fleS/flrB,flgA,flgB,flgC,flgD,flgE,flgF,flgG,flgH,flgI,flgJ,flgK,flgL,flgM,flgN,flgO,flgP,flgT,flhA,flhB,flhF,fliA,fliD,fliE,fliF,fliG,fliH,fliI,fliJ,fliK,fliL,fliM,fliN,fliO,fliP,fliQ,fliR,fliS,flrA,hcp-2,hlyA,icmF/vasK,luxS,motA,motB,motX,motY,mshB,mshC,mshD,mshE,mshF,mshG,mshH,mshI,mshJ,mshK,mshL,mshM,mshN,rtxB,rtxC,rtxD,vasA,vasB,vasC,vasD,vasE,vasF,vasH,vasI,vasJ,vasL,vgrG-2,vipA/mglA,vipB/mglB,
C_21	108	VCA0109,VCA0122,VPA1340,VPA1353,cheA,cheB,cheR,cheV,cheW,cheY,cheZ,clpB/vasG,cqsA,flaA,flaB,flaC,flaD,flaE,flaG,flaI,fleN,fleR/flrC,fleS/flrB,flgA,flgB,flgC,flgD,flgE,flgF,flgG,flgH,flgI,flgJ,flgK,flgL,flgM,flgN,flgO,flgP,flgT,flhA,flhB,flhF,fliA,fliD,fliE,fliF,fliG,fliH,fliI,fliJ,fliK,fliL,fliM,fliN,fliO,fliP,fliQ,fliR,fliS,flrAhcp-2,hlyA,icmF/vasK,luxS,motA,motB,motX,motY,mshB,mshC,mshD,mshE,mshF,mshG,mshH,mshI,mshJ,mshK,mshL,mshM,mshN,rtxA,rtxB,rtxC,rtxD,vasA,vasB,vasC,vasD,vasE,vasF,vasH,vasI,vasJ,vasL,vcrD2,vgrG-2,vipA/mglA,vipB/mglB,vopB2,vopD2,vscC2,vscN2,vscR2,vscS2,vscT2,vscU2,
C_22	97	VCA0109,VCA0122,cheA,cheB,cheR,cheV,cheW,cheY,cheZ,clpB/vasG,cqsA,flaA,flaB,flaC,flaD,flaE,flaG,flaI,fleN,fleR/flrC,fleS/flrB,flgA,flgB,flgC,flgD,flgE,flgF,flgG,flgH,flgI,flgJ,flgK,flgL,flgM,flgN,flgO,flgP,flgT,flhA,flhB,flhF,fliA,fliD,fliE,fliF,fliG,fliH,fliI,fliJ,fliK,fliL,fliM,fliN,fliO,fliP,fliQ,fliR,fliS,flrA,hcp-2,hlyA,icmF/vasK,luxS,motA,motB,motX,motY,mshB,mshC,mshD,mshE,mshF,mshG,mshH,mshI,mshJ,mshK,mshL,mshM,mshN,rtxA,rtxB,rtxC,rtxD,vasA,vasB,vasC,vasD,vasE,vasF,vasH,vasI,vasJ,vasL,vgrG-2,vipA/mglA,vipB/mglB,

C_23	97	VCA0109,VCA0122,cheA,cheB,cheR,cheV,cheW,cheY,cheZ,clpB/vasG,cqsA,flaA,flaB,flaC,flaD,flaE,flaG,flaI,fleN,fleR/flrC,fleS/flrB,flgA,flgB,flgC,flgD,flgE,flgF,flgG,flgH,flgI,flgJ,flgK,flgL,flgM,flgN,flgO,flgP,flgT,flhA,flhB,flhF,fliA,fliD,fliE,fliF,fliG,fliH,fliI,fliJ,fliK,fliL,fliM,fliN,fliO,fliP,fliQ,fliR,fliS,flrA,hcp-2,hlyA,icmF/vasK,luxS,motA,motB,motX,motY,mshB,mshC,mshD,mshE,mshF,mshG,mshH,mshI,mshJ,mshK,mshL,mshM,mshN,rtxA,rtxB,rtxC,rtxD,vasA,vasB,vasC,vasD,vasE,vasF,vasH,vasI,vasJ,vasL,vgrG-2,vipA/mglA,vipB/mglB,
C_24	108	VCA0109,VCA0122,VPA1340,VPA1353,cheA,cheB,cheR,cheV,cheW,cheY,cheZ,clpB/vasG,cqsA,flaA,flaB,flaC,flaD,flaE,flaG,flaI,fleN,fleR/flrC,fleS/flrB,flgA,flgB,flgC,flgD,flgE,flgF,flgG,flgH,flgI,flgJ,flgK,flgL,flgM,flgN,flgO,flgP,flgT,flhA,flhB,flhF,fliA,fliD,fliE,fliF,fliG,fliH,fliI,fliJ,fliK,fliL,fliM,fliN,fliO,fliP,fliQ,fliR,fliS,flrA,hlyA,icmF/vasK,luxS,motA,motB,motX,motY,mshB,mshC,mshD,mshE,mshF,mshG,mshH,mshI,mshJ,mshK,mshL,mshM,mshN,rtxA,rtxB,rtxC,rtxD,vasA,vasB,vasC,vasD,vasE,vasF,vasH,vasI,vasJ,vasL,vcrD2,vgrG-2,vipA/mglA,vipB/mglB,vopB2,vopD2,vscC2,vscN2,vscQ2,vscR2,vscS2,vscT2,vscU2,
C_25	95	VCA0109,VCA0122,cheA,cheB,cheR,cheV,cheW,cheY,cheZ,clpB/vasG,cqsA,flaA,flaB,flaC,flaD,flaE,flaG,flaI,fleN,fleR/flrC,fleS/flrB,flgA,flgB,flgC,flgD,flgE,flgF,flgG,flgH,flgI,flgJ,flgK,flgL,flgM,flgN,flgO,flgP,flgT,flhA,flhB,flhF,fliA,fliD,fliE,fliF,fliG,fliH,fliI,fliJ,fliK,fliL,fliM,fliN,fliO,fliP,fliQ,fliR,fliS,flrA,hcp-2,hlyA,icmF/vasK,luxS,motA,motB,motX,motY,mshB,mshC,mshD,mshE,mshF,mshG,mshH,mshI,mshJ,mshK,mshL,mshM,mshN,rtxB,rtxC,rtxD,vasA,vasB,vasC,vasD,vasE,vasF,vasH,vasI,vasJ,vasL,vipA/mglA,vipB/mglB,
C_26	110	VCA0109,VCA0122,VPA1340,VPA1353,cheA,cheB,cheR,cheV,cheW,cheY,cheZ,clpB/vasG,cqsA,flaA,flaB,flaC,flaD,flaE,flaG,flaI,fleN,fleR/flrC,fleS/flrB,flgA,flgB,flgC,flgD,flgE,flgF,flgG,flgH,flgI,flgJ,flgK,flgL,flgM,flgN,flgO,flgP,flgT,flhA,flhB,flhF,fliA,fliD,fliE,fliF,fliG,fliH,fliI,fliJ,fliK,fliL,fliM,fliN,fliO,fliP,fliQ,fliR,fliS,flrA,hcp-2,hlyA,icmF/vasK,luxS,motA,motB,motX,motY,mshB,mshC,mshD,mshE,mshF,mshG,mshH,mshI,mshJ,mshK,mshL,mshM,mshN,rtxA,rtxB,rtxC,rtxD,vasA,vasB,vasC,vasD,vasE,vasF,vasH,vasI,vasJ,vasL,vcrD2,vgrG-2,vipA/mglA,vipB/mglB,vopB2,vopD2,vscC2,vscJ2,vscN2,vscQ2,vscR2,vscS2,vscT2,vscU2,
C_27	95	VCA0109,VCA0122,cheA,cheB,cheR,cheV,cheW,cheY,cheZ,clpB/vasG,flaA,flaB,flaC,flaD,flaE,flaG,flaI,fleN,fleR/flrC,fleS/flrB,flgA,flgB,flgC,flgD,flgE,flgF,flgG,flgH,flgI,flgJ,flgK,flgL,flgM,flgN,flgO,flgP,flgT,flhA,flhB,flhF,fliA,fliD,fliE,fliF,fliG,fliH,fliI,fliJ,fliK,fliL,fliM,fliN,fliO,fliP,fliQ,fliR,fliS,flrA,hcp2,hlyA,icmF/vasK,luxS,motA,motB,motX,motY,mshB,mshC,mshD,mshE,mshF,mshG,mshH,mshI,mshJ,mshK,mshL,mshM,mshN,rtxB,rtxC,rtxD,vasA,vasB,vasC,vasD,vasE,vasF,vasH,vasI,vasJ,vasL,vgrG-2,vipA/mglA,vipB/mglB,
C_28	96	VCA0109,VCA0122,cheA,cheB,cheR,cheV,cheW,cheY,cheZ,clpB/vasG,cqsA,flaA,flaB,flaC,flaD,flaE,flaG,flaI,fleN,fleR/flrC,fleS/flrB,flgA,flgB,flgC,flgD,flgE,flgF,flgG,flgH,flgI,flgJ,flgK,flgL,flgM,flgN,flgO,flgP,flgT,flhA,flhB,flhF,fliA,fliD,fliE,fliF,fliG,fliH,fliI,fliJ,fliK,fliL,fliM,fliN,fliO,fliP,fliQ,fliR,fliS,flrA,hcp2,hlyA,icmF/vasK,luxS,motA,motB,motX,motY,mshB,mshC,mshD,mshE,mshF,mshG,mshH,mshI,mshJ,mshK,mshL,mshM,mshN,rtxB,rtxC,rtxD,vasA,vasB,vasC,vasD,vasE,vasF,vasH,vasI,vasJ,vasL,vgrG-2,vipA/mglA,vipB/mglB,
F_1	16	cheW,cheY,flaD,fleN,flgB,flgC,flgD,flgG,flgO,fliG,fliM,fliN,fliS,hcp-2,luxS,vipB/mglB
F_2	16	cheW,cheY,flaD,fleN,flgB,flgC,flgD,flgG,flgO,fliG,fliM,fliN,fliS,hcp-2,luxS,vipB/mglB
P_1	45	VP1611,VPA0450,cheY,exsA,exsD,flgB,fliG,sycN,tlh,tyeA,vcrD,vcrG,vcrH,vcrR,vcrV,vecA,virG,vopB,vopD,vopN,vopQ,vopR,vopS,vpadF,vscB,vscC,vscD,vscF,vscG,vscH,vscI,vscJ,vscK,vscL,vscN,vscO,vscP,vscQ,vscR,vscS,vscT,vscU,vscX,vscY,vxsC
P_2	44	VP1611,VPA0450,cheY,exsA,exsD,flgB,fliG,sycN,tlh,tyeA,vcrD,vcrG,vcrH,vcrR,vcrV,vecA,virG,vopB,vopD,vopN,vopQ,vopR,vopS,vscB,vscC,vscD,vscF,vscG,vscH,vscI,vscJ,vscK,vscL,vscN,vscO,vscP,vscQ,vscR,vscS,vscT,vscU,vscX,vscY,vxsC
P_3	44	VP1611,VPA0450,cheY,exsA,exsD,flgB,fliG,sycN,tlh,tyeA,vcrD,vcrG,vcrH,vcrR,vcrV,vecA,virG,vopB,vopD,vopN,vopQ,vopR,vopS,vscB,vscC,vscD,vscF,vscG,vscH,vscI,vscJ,vscK,vscL,vscN,vscO,vscP,vscQ,vscR,vscS,vscT,vscU,vscX,vscY,vxsC
V_1	9	flaA,cheW,cheY,flgB,flgC,fliG,ompU,rtxB,rtxC
V_2	9	flaB,cheW,cheY,flgB,flgC,fliG,ompU,rtxB,rtxC
V_3	9	flaC,cheW,cheY,flgB,flgC,fliG,ompU,rtxB,rtxC
V_4	8	flaD,cheW,cheY,flgC,fliG,ompU,rtxB,rtxC
V_5	9	flaE,cheW,cheY,flgB,flgC,fliG,ompU,rtxB,rtxC
V_6	9	flaG,cheW,cheY,flgB,flgC,fliG,ompU,rtxB,rtxC
V_7	9	flaI,cheW,cheY,flgB,flgC,fliG,ompU,rtxB,rtxC
V_8	9	fleN,cheW,cheY,flgB,flgC,fliG,ompU,rtxB,rtxC
V_9	9	fleR/flrC,cheW,cheY,flgB,flgC,fliG,ompU,rtxB,rtxC

V_10	9	fleS/flrB,cheW,cheY,flgB,flgC,fliG,ompU,rtxB,rtxC
V_11	-	
V_12	9	flgB,cheW,cheY,flgB,flgC,fliG,ompU,rtxB,rtxC
V_13	9	flgC,cheW,cheY,flgB,flgC,fliG,ompU,rtxB,rtxC
